# Applying the Consensual Method of Estimating Poverty in a Low Income African Setting

**DOI:** 10.1007/s11205-014-0819-z

**Published:** 2014-12-05

**Authors:** Shailen Nandy, Marco Pomati

**Affiliations:** 1 Townsend Centre for International Poverty Research, School for Policy Studies, University of Bristol, 8 Priory Road, Bristol, BS8 1TZ UK; 2 Cardiff University School of Social Sciences, Glamorgan Building, King Edward VII Avenue, Cardiff, CF10 3WT UK

**Keywords:** Poverty, Deprivation, Consensual approach, Socially perceived necessities, Benin, West Africa

## Abstract

We present the first study of multidimensional poverty in Benin using the consensual or socially perceived necessities approach. There is a remarkable level consensus about what constitutes the necessities of life and an adequate standard of living. Following Townsend’s concept of relative deprivation, we show how social consensus provides the basis for a reliable and valid index of multiple deprivation, which can be used to reflect multidimensional poverty. We discuss the issue of adaptive preferences, which has previously been used to criticise the consensual approach, and provide evidence to contest the claim that the poor adjust their aspirations downwards.

## Introduction

In the run up to the 2015 target date for the Millennium Development Goals (MDGs), there are an increasing number of claims that the first MDG (to reduce by half the proportion of people living in extreme poverty) has been met ahead of schedule, even for the poorest region of the world, sub-Saharan Africa (Chandy and Gertz 2011; Pinkovskiy and Sala-I-Martin 2010; United Nations 2012). While heated debate continues about the choice of indicator being used to monitor progress (the World Bank’s commonly referred to “Dollar a Day”), and its suitability to reflect adequately and appropriately the needs of poor people around the world (Chen and Ravallion 2008; Gordon 2002; Reddy and Pogge 2008) even older discussions, on how poverty should be conceptualised, assessed and measured, continue among academics, policy makers and others interested in assessing people’s living conditions.

There has in recent years been a convergence of opinions of what constitutes poverty, in that few (if any) would claim it is simply a low level of income; rather, it appears there is general consensus that poverty is relative to time and place, and that absolutist notions of subsistence-based poverty lines are no longer tenable in the twenty first century, as peoples’ needs have expanded along with their rights and entitlements to freedom from starvation and destitution. International conventions, from the Universal Declaration of Human Rights to the UN Convention on the Rights of the Child enshrine people’s rights to an adequate standard of living, to assistance in times of need, to basic social security (Townsend 2009) and, most recently, to the ILO-supported minimum social protection floor.^1^ International definitions of poverty, including that adopted at the 1995 World Summit on Social Development, increasingly highlight non-monetary dimensions or aspects of poverty, which measures and indicators are required to reflect. This paper adopts the consensual approach to poverty measurement, a method which has been used to great effect in a number of high and middle income countries, and applies it to a low-income country (Benin). In doing so, we set out the conceptual framework and explain the consensual approach. We then demonstrate its applicability to a low-income country, and how consensus can be ascertained in a socially, economically, culturally and linguistically diverse country like Benin. Key results are presented, along with a discussion of the issue of adaptive preferences and the implications of the approach for researchers interested in pursuing similar work in other countries.

## Conceptualising ‘Need’ and the Consensual Approach to Poverty

As previously mentioned, there is now consensus that poverty no longer constitutes simply a low income, and that non-monetary aspects or dimensions of poverty are also important to consider. Related to this are a number of changes in the way people’s needs are conceptualised and ascertained. Early poverty surveys (e.g. those by Rowntree in the early 1900s) focused on more absolutist, subsistence needs, such as having enough money to buy sufficient food to prevent starvation, clothing and shelter. Subsequent researchers, like Peter Townsend, realised such minimalist thresholds were insufficient, and instead developed ideas around the concept of relative deprivation. For Townsend,

“Individuals, families and groups in the population can be said to be in poverty when they lack the resources to obtain the types of diet, participate in the activities and have the living conditions and amenities which are customary, or are at least widely encouraged or approved, in the societies to which they belong. Their resources are so seriously below those commanded by the average individual or family that they are, in effect, excluded from ordinary living patterns, customs and activities” (Townsend 1979: 31).

According to Townsend people’s needs have to be understood in terms of contemporary living standards and social customs, and thus, as these change over time, so too do measures and indicators of poverty. “*Any conception of poverty as ‘absolute’*…” he argues, is “…*inappropriate and misleading*…” (Townsend 1979: 38). Linked to this was a recognition that poverty measures also needed to reflect people’s social needs rather than just their base physical or material needs (food, clothing, shelter), or at least reflect them better than they had been done previously. This resulted in items related to peoples’ social participation (e.g. gift giving, commemorating special occasions, customary social obligations, etc.) being added to poverty surveys in a number of countries.

A frequent criticism of poverty surveys is that they too often rely on the opinions of well-remunerated experts (academics, social workers, etc.), and rarely incorporate the opinions of those with a lived experience of poverty—i.e. poor people themselves—or the wider public (Citro and Michael 1995). Such concerns led Mack and Lansley to develop the Consensual Approach to poverty, which makes the opinions of the general public a central part in both the definition and measurement of poverty. Building on Townsend’s earlier work and his concept of relative deprivation, Mack and Lansley developed and refined methods which asked the general public about their views on what constituted an acceptable minimum standard of living. Focus group discussions (Pantazis et al. 2006a) were used to see what types of items (e.g. warm winter coat) and activities (e.g. celebrate special occasions) people thought no one should have to go without due to a lack of money—i.e. were essential and necessary. Those items which a majority of respondents (i.e. 50 % or more) believed to be necessary were then taken forward and respondents asked (either in the same survey, or in a separate survey as has been done most recently in the UK 2012 Poverty and Social Exclusion Survey) if they had or did not have the items, and if they lacked them was this due to choice (i.e. did not want) or a lack of resources (i.e. could not afford). The items a majority of people thought necessities formed the basis of a deprivation index, on which respondents lacking items considered necessities because they could not afford them, scored (for each item) a 1 (deprived) or 0 (not deprived). Townsend’s work showed that multiple deprivation and poverty are highly correlated, and that below a certain level of income (or resources), the number of deprivations experienced increases rapidly (Gordon 2006; Townsend 1979). It was at this point, or kink, of between two and four deprivations (depending on the study) that a threshold could be set to divide the ‘poor’ from the ‘not poor’ (or the deprived from the not deprived). As Gordon noted, when setting out criteria for identifying an objective poverty line, this should be the point “*that maximises differences between the two groups (‘poor’ and ‘not poor’) and minimises the differences within the two groups (‘poor’ and ‘not poor’)”* (Gordon 2006: 39).

Over the last few decades, the consensual approach has been used to examine relative poverty in a number high-income countries, including Great Britain (Gordon and Pantazis 1997a, Mack and Lansley 1985; Pantazis et al. 2006a), Northern Ireland (Hillyard et al. 2003), Belgium (Van Den Bosch 2001), Sweden (Halleröd 1994, 1995), Finland (Halleröd et al. 2006), Japan (Abe and Pantazis 2013) and Australia (Saunders and Wong 2011). More recently, researchers have begun to use the method in middle income and low income countries, including in Bangladesh (Ahmed 2007), Vietnam (Davies and Smith 1998), Mali (Nteziyaremye and Mknelly 2001), Tanzania (Kaijage and Tibaijuka 1996), Zimbabwe (Mtapuri 2011) and South Africa (Noble et al. 2004; Wright 2008). In each instance the list of items/activities has been modified to reflect local conditions and customs, and in each the method has been found to be effective and reliable. Here, we report on our use of the method in the context of Benin.

## Data and Methods

The data used in this paper were provided in the 2006 Demographic and Household (DHS) Survey for Benin. DHS data are regularly used by organisations like the United Nations and researchers to assess people’s living conditions in low income countries (Corsi et al. 2012; Gordon et al. 2003; Vaessen 1996).

Benin, located on the west coast of Africa between Togo and Nigeria, had in 2006 an estimated population of between 8 and 9 million. It is classed by the World Bank as a low-income economy, and in 2011 was ranked 166th out of 187 countries by the United Nations Development Programme’s Human Development Index. In 2005, the year closest to the data used in the paper, life expectancy at birth was 53.9 years, the mean number of years of schooling was 2.9 and the gross national income per capita (adjusted for purchasing power parity) was $1,340 (UNDP 2013). In 2007, around one-third of the population had incomes below the national poverty line. As with most countries in Africa, the population of Benin is socially and culturally heterogeneous; over a dozen distinct languages are spoken across the many different tribal and ethnic groups, which follow a mix of Muslim, Christian and African Traditional religions.

The 2006 DHS for Benin provides data collected separately though the Integrated Modular Survey on Household Living Conditions, run by the National Institute of Statistics and Economic Analysis. This module ascertained respondents’ opinions about what items/activities they considered to be necessary for a decent standard of living (INSAE 2007). Questionnaires were translated into the main languages of Benin (French, Adja, Bariba, Fon, Dendi, Ditamari and Yoruba), and interviews conducted mainly in French but also in Adja, Bariba, Fon, Dendi, Ditamari, and Yoruba.^2^ Respondents were asked about their perceptions of necessities and also the degree to which they felt these needs were met for them and their families. Questions were asked about a range of items, covering food, clothing, housing, health/hygiene, transportation, education and leisure, work and relationships.

The survey used a multi-stage stratified sample, and was administered to 17,511 households, with one adult respondent per household. The sample reflected the roughly 40:60 urban-rural split in population and was representative at sub-national level. Full details of the sample are available from the national DHS report.^3^

Respondents were asked, from a list of 26 items/activities (covering food, clothing, housing, transport, etc) which they considered to be essential (“*indispensable*” in French) to have to achieve a decent standard of living.^4^ Included on the list were:^5^

Food and clothing items

1. Eat three meals a day every day
2. Eat cereals or tubers daily
3. Eat vegetables every day
4. Eating meat or fish every day
5. A good meal on festivities/celebrations (Sunday, ceremony, etc.)
6. Have a change of clothes (at least two)
7. Having several pairs of shoes (at least two)

Housing items

8. Having a home (as a tenant or owner)
9. Having spacious housing (rented or not)
10. Have access to drinking water
11. Have access to electricity
12. Have furniture (tables and beds) in the house
13. Able to buy cleaning products (soap, wax, etc.).

Health, body care items

14. Able to heal when you are sick
15. Able to take care of one’s body (soap, hairdresser, etc.).

Work items

16. Having a stable and long-term job
17. Working day and night

Transport items

18. Able to take the bus (or equivalent) to work
19. Able to take a taxi if necessary (emergency)
20. Having a personal means of transport (motorcycle, bicycle)

Education, leisure and other

21. The ability to send children to school
22. Taking a vacation once a year (travel)
23. Having a radio
24. Able to buy a television
25. Able to offer gifts when necessary
26. Control one’s fertility

Respondents were then asked (for a sub-set of the original items) the degree to which they felt their own and their households’ needs were satisfied.^6^ The sub-set of items/activities about which follow up questions were asked included the number of meals per day, daily consumption of cereal/root vegetables, daily consumption of vegetables, daily consumption of meat or fish, meals on holidays and special occasions, clothing, shoes, the quality of respondent’s homes, access to drinking water and electricity, furniture in the home, availability of cleaning products, access to health facilities, medicines for illness, personal hygiene products (e.g. soap), the means of transport used, education for Children, leisure (or holiday), relationships with family and friends, and being able to provide assistance to parents in time of difficulty.

Studies like the 2012 Poverty and Social Exclusion Survey in the UK follow a two-stage process; people’s perceptions about what items/activities they consider necessities are ascertained in one survey, and a second survey (on a different sample) asks respondents if they have/do the items/activities asked in the previous survey, and if they lack or do not do them is it out of choice or because they cannot afford to own/do them. Those reporting they lack an item (which a majority at the first stage considered a necessity) because they cannot afford it, are considered deprived. Others doing similar work, such as Saunders et al. (2007) in Australia, used a single survey to ascertain both opinions about necessities and whether or not people lacked them.^7^ This paper follows the approach taken by Saunders et al. (2007), in using data where both sets of information were collected in a single survey. We consider only responses which state items/activities as ‘essential’ (i.e. “*indispensable*”) to be conceptually equivalent as ‘Necessity’ in other consensual studies, as counting items defined as either “*plutot nécessaire*” (quite necessary) or “*indispensable*” (essential) would result in almost universal agreement across all items on the list. The difference between quite necessary and essential was also suggested by the questionnaire wording which asked respondents if the item was: (1) essential or (2) quite necessary (3) not needed. Quite necessary was therefore used as the mid-point between essential and not needed, and we therefore decide to consider only the items considered essential. The adoption of this stricter threshold ensures we err on the side of caution, and are more certain that items counted as essential are really considered such by respondents.

Relative risk ratios and their 95 % Confidence Intervals (CI) were computed to show whether differences between groups of respondents were statistically significant, and also the size and direction of these differences (Morris and Gardner 1988). Relative risk ratios show the probability, or risk, of one group thinking an item to be essential compared to another group. A relative risk of 2 means twice the risk, a risk of 0.5 implies half the risk, etc. Where confidence intervals cross 1 means there is no difference between the two groups (Gordon 2013).

## Results

Table 1 shows which items and activities respondents in Benin consider to be essential for a decent standard of living. Most (over 80 %) consider important basics, such as having access to drinking water, to care when sick and having steady work as essential. A majority also believe that (among other things) having three meals a day, being able to send children to school, having access to electricity and a form of transportation are all essential. The table also shows (final column) the proportion of respondents reporting that their needs for each item were “*not at all satisfied*”; nearly half (46 %) of people felt their need for electricity was not met, around a third (31 %) felt they lacked access to a mode of transport, and over a quarter lacked access to drinking water (i.e. were more than likely to be using unimproved or unsafe sources of water, like rivers/streams). A total of 22 out of the 26 items were considered by a majority of respondents to be essential. Of these 22, need satisfaction data were available for 16, and it is these which form the basis of the deprivation index, where responses reporting that needs were “*not at all satisfied*” counted as deprived (scoring 1) and other responses scoring 0. Scores were summed to make a final deprivation index, with a maximum score of 16 and a minimum score of 0.

**Table 1:** List of items respondents considered essential

	Essential (%)	More or less necessary (%)	No (%)	% Respondents reporting needs “not at all satisfied”
Need to have access to drinking water	84	15	0	26
Need to take care of oneself when sick	84	16	0	19
Having a stable and long-term job	82	18	1	^a^
Need to be able to send children to school	79	20	1	13
Need to have access to electricity	77	22	2	46
Need to have three meals per day	74	24	2	7
Need to have a house	71	24	5	16
Need to have a radio	71	28	1	^a^
Need to have mode of transportation	68	30	2	31
Need to take of own body (soap, barber etc.)	67	32	1	11
A good meal on festivities/celebrations (Sunday, ceremony, etc.)	64	33	4	7
Need to have personal care products	62	36	2	15
Need to have tables and beds	62	36	2	36
Have a change of clothes (at least two)	61	36	2	10
Need to have a spacious house	59	37	4	^a^
Need to be able to buy a television	59	38	4	^a^
Need to have several sets of shoes	58	38	4	10
Need to have meat or fish every day	57	36	7	10
Need to be able to take a taxi	56	42	2	^a^
Need to have birth control	55	37	8	^a^
Need to have cereals or food made from roots or tubers every day	51	37	12	5
Need to take vacation	51	43	6	22
Need to be able to take the bus	45	46	9	^a^
Need to be able to buy presents when needed	44	51	5	^a^
Need to have vegetables every day	43	40	17	5
Need to work day and night	17	24	60	^a^

*Source*: Calculated from Benin DHS 2006; N = 17,483

^a^No follow up question regarding need satisfaction asked

### Establishing Consensus

The Consensual Approach firstly identifies publicly perceived necessities and then proceeds to find out who lacks these, so to move confidently from the first to the second stage it is important to demonstrate consensus about the list of items in the deprivation index. While there is no reason to assume people will agree on what items take priority over others^8^ it is important to establish horizontal agreement, i.e. that different demographic groups all agree that a particular item is essential or a necessity.

One way to demonstrate this is through the use of heat maps, where respondent’s answers are shaded; items receiving a higher prevalence of positive responses (e.g. thinking that access to drinking water is essential) are shaded darker, with those with lower prevalence a lighter shade. Table 2 shows the proportion of respondents considering an item essential, by their age and gender. Tables 5, 6 and 7 show the degree of consensus by respondent’s level of education and migrant status, religion and ethnicity, and (for the sake of conciseness) are provided in Appendix “2”. What each table clearly shows is the high degree of horizontal consensus; i.e. what younger respondents think essential is very similar to what older respondents report; what women think are essential are also likely to be thought essential by men, etc. What (slight) differences there may be can be explained on a case for case basis (e.g. religious or cultural prohibition).

**Table 2:**
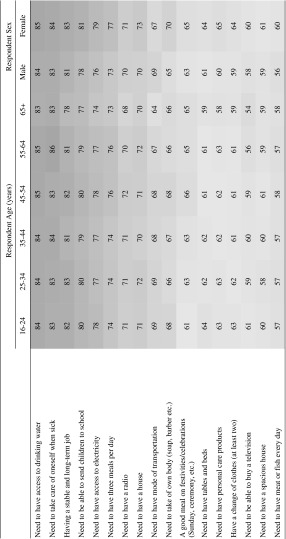

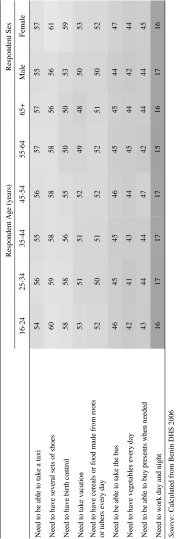
Heat map of attitudes to items considered “essential”, by respondent age and sex (%). (Color figure online)

### Item Validity

Table 1 showed that a total of 22 items out of 26 asked about were considered by more the 50 % of respondents to be “essential”. Of these 22, follow-up questions were asked as to whether respondents felt their needs were met with regards 16 of the items. Table 1 also shows the proportion of respondents reporting that their needs were not at all satisfied. So while 84 % of people thought having access to water was essential, 26 % felt this need was not satisfied at all, suggesting a high level of deprivation of a socially perceived necessity (water). The 16 items regarding need-satisfaction form the basis of a deprivation index: respondents score a 1 for each item of which they are deprived. Respondents could have a minimum score of 0 and a maximum of 16.

When constructing deprivation indices, it is important that each item be both a reliable and valid measure of poverty (Gordon 2006). The overall reliability of the scale is discussed in Sect. 4.3; here we show how the validity of each item was tested against four measures known to relate to poverty. Four different validators were used:

1. Respondent’s evaluations of their household income status: the probability of being deprived for those who thought household income status “difficult” was compared to the probability of those who thought their household income status either “good” or “more or less OK”;
2. Respondent’s evaluations of their current financial situation: the probability of being deprived for those going into debt was compared to the probability for those who were able to save either a little or a reasonable amount;
3. Respondent’s evaluations of the stability of their household income: the probability of being deprived for those considering their household income unstable compared with those for whom household income was considered stable;
4. Respondent’s quintile on the DHS household wealth index: the probability of being deprived for the bottom 20 % was compared to the top 20 %.

In each of the 64 instances (i.e. 16 items × 4 validators), the probability of being deprived was significantly greater for those known to be disadvantaged compared with those who were not. So, for example, with regards validator 1, respondents who felt their household income status to be ‘difficult’ were nearly 15 times more likely to feel that their needs for the requisite number of meals each day were not at all met, compared to those whose household income status was reported as good or more or less OK. Results for each validator are presented in Appendix “3”. There is clear face validity for the index, given that the items which go into making it up are those which relate to people’s everyday living conditions and their needs for clothing, food, health care and transport.

**Fig. 1:**
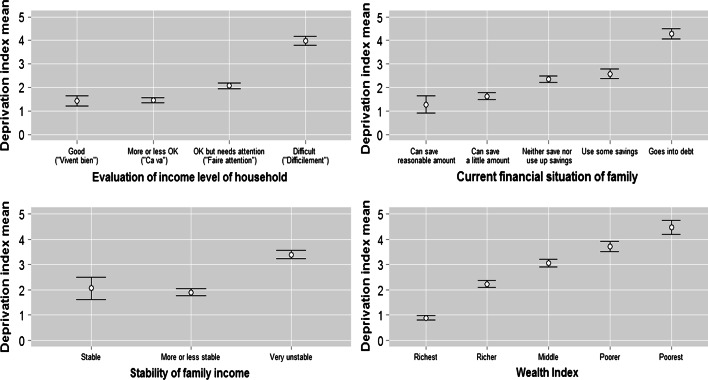
Testing scale validity. *Source*: Calculated from Benin DHS 2006

### Scale Reliability

Scale reliability was tested using Cronbach’s Alpha, and was found to be high, with an alpha of 0.885 (Table 3). This can be interpreted as the average correlation between this set of 16 questions (i.e. relating to items in the deprivation index) and all other sets of deprivation questions of equal length (i.e. in this instance, 16 items) (Nunnally 1981; Devellis 2003).

**Table 3:** Scale reliability

	Scale mean if item deleted	Scale variance if item deleted	Corrected item-total correlation	Cronbach’s alpha if item deleted
Satisfied with number of meals every day	12.22	11.57	0.53	0.880
Satisfied with consumption of cereals and tubers every day	12.21	11.69	0.52	0.880
Satisfied with consumption of meat or fish every day	12.25	11.37	0.54	0.879
Satisfied with meals on Sundays and Holidays	12.23	11.49	0.55	0.879
Satisfied with clothing	12.26	11.17	0.64	0.875
Satisfied with shoes	12.26	11.14	0.64	0.875
Satisfied with housing	12.31	11.05	0.56	0.877
Satisfied with availability of drinking water	12.42	10.96	0.48	0.882
Satisfied with availability of electricity	12.62	10.62	0.51	0.881
Satisfied with furniture in the house	12.52	10.48	0.59	0.877
Satisfied with self-care products in the house	12.31	10.95	0.62	0.875
Satisfied with care in case of sickness	12.34	10.85	0.60	0.876
Satisfied with cleanliness/personal hygiene	12.27	11.13	0.62	0.876
Satisfied with availability of transport	12.47	10.81	0.50	0.881
Satisfied with availability of leisure	12.38	11.06	0.47	0.881
Satisfied with education for children	12.29	11.26	0.51	0.879
Reliability statistics				
Cronbach’s alpha				
0.885				
Scale statistics				
Mean	Variance	SD	No. of items	
13.16	12.53	3.54	16	

*Source*: Calculated from Benin DHS 2006

### Scale Validity

The validity of a scale can be assessed by seeing if it exhibits statistically significant associations with a set of independent variables known to be correlated with poverty (Pantazis et al. 2006b). For example, it would be expected, from Townsend’s theory of relative deprivation and Mack and Lansley’s concept of “consensual poverty”, that someone who is ‘deprived’ would also be more likely to consider her/himself to be subjectively poor (Bradshaw and Finch 2003), to have a lower level of household resources or assets, or have an unstable income or household financial situation. We tested the deprivation index for Benin against the four validators described above, each of which we know are correlated with poverty. In each instance, the mean deprivation score was highest (i.e. signifying a higher level of deprivation) for respondents identified by the validators as being in the worst circumstances (Fig. 1).

### Prevalence of Deprivation in Benin

**Fig. 2:**
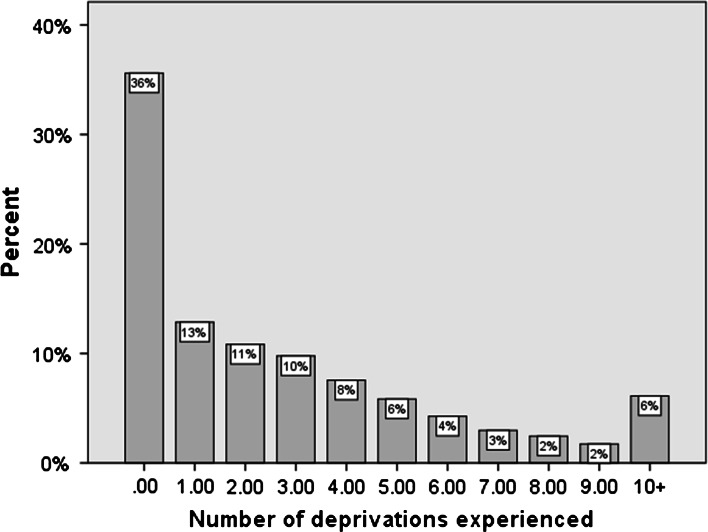
Numbers of deprivations experienced. *Source*: Calculated from Benin DHS 2006. N = 17,511. (Color figure online)

Having explained how items were identified and the deprivation index or scale developed, Fig. 2 shows the pattern of deprivations across the whole sample. While just over a third (36 %) of respondents reported that their needs with regards the 16 items on the deprivation index were met to one degree or another, and thus were classified as not experiencing any deprivations, around two-thirds felt their needs for at least one item were not at all satisfied. The pattern is as one would expect, with the proportion of respondents deprived decreasing as the numbers of deprivations increase. Around 6 % of respondents were deprived of ten or more items.

### Poverty/Deprivation Threshold

Townsend (1979) showed there is a clear relationship between the resources people have, and their ability to avoid the consequences of poverty, deprivation. Previous studies (Gordon and Pantazis 1997c) of poverty using the consensual approach have used household income as a measure of resources which people use to protect or cushion themselves against deprivation, and each show there is a point on the distribution below which the experience of multiple deprivations increases much more rapidly. Below this set level of resources (income or other), people are no longer able to satisfy their basic needs, and the result is multiple deprivation and undeniable poverty.

**Fig. 3:**
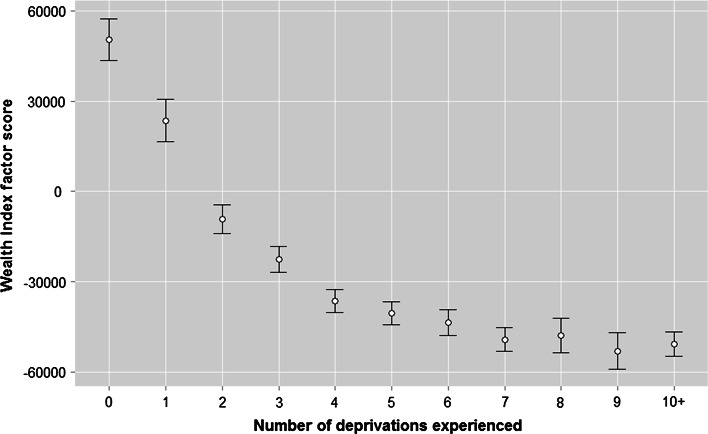
Mean wealth index factor score by number of deprivations experienced. *Source*: Calculated from Benin DHS 2006

The DHS data for Benin do not collect data on household income or expenditure so we cannot do a similar exercise to identify the kink or threshold. What we can do, however, is use the raw scores of the DHS wealth index (Rutstein and Johnson 2004) as a proxy for household resources. The asset index uses information about household assets (e.g. ownership of land, vehicles, consumer durables, etc) and the provision of basic services (e.g. access to electricity, piped water, sanitation) to provide households with a score on a continuous scale. These scores can be used to rank households in a distribution, or grouped into categories, like quintiles. As one would expect, there is a clear relationship between the asset index score and deprivation, with respondents experiencing no deprivations having significantly higher asset index scores (Fig. 3). Below a certain point of household resources (on the y-axis), the number of deprivations experienced (on the x-axis) increases sharply, and this is where one would consider setting a poverty line or threshold (if one was using income on the y-axis). In this instance, based on a visual assessment, we would consider respondents experiencing four or more deprivations to be below the asset-index based poverty line, and would consider all such households as poor. ANOVA and Logistic Regression analyses proposed by Gordon (2006) to identify thresholds in the relationship between income and deprivation suggest a threshold of three or more; however, given that income and the Wealth Index are conceptually very different measures, we err on the side of caution and we set the poverty threshold at four or more deprivations. Such households accounted for just under one-third (31 %) of households in Benin, which is similar to the proportion of households living below the national poverty line of Benin, of 33 %;^9^ were the threshold set higher, at 5+ deprivations, 23 % of households would be classed as poor. Just over a third (36 %) of households did not experience any deprivations.

### Country Profile of Multiple Deprivation

Table 4 shows how multiple deprivation is patterned across different geographic regions and socio-cultural groups in Benin. Focussing solely on those experiencing 4+ deprivations, prevalence rates in rural areas are twice those of urban areas. There are considerable regional differences, with nearly half of households in Collines experiencing 4+ deprivations; this is in contrast to one in eight households in Littoral. As expected, the prevalence of multiple deprivation is highest for those in the poorest wealth index quintile, with a clear gradient apparent. The fact that 7 % of households in the top quintile report experiencing 4+ deprivations suggests the wealth index is classifying some deprived responds as relatively wealthy, which we consider problematic. Researchers have questioned the methods used to create the wealth index and its ability to make meaningful or reliable comparisons between countries or over time (Falkingham and Namazie 2002; Howe et al. 2008). Our analysis shows the wealth index may identify as wealthy some very deprived households. That said, in low income countries like Benin, where deprivation with regards some basic needs (e.g. access to electricity, or to safe drinking water) is generally high, such a finding is not entirely unexpected.

**Table 4:** The patterning of multiple deprivation in Benin, 2006 (%)

	No deprivations	1-3 deprivations	4+ deprivations
Urban–rural			
Benin	36	34	31
Rural	28	33	39
Urban	47	34	19
Region			
Collines	16	36	48
Atacora	35	23	43
Plateau	26	32	42
Zou	29	35	37
Quémé	29	39	33
Couffo	36	31	33
Atlantique	34	34	32
Mono	46	25	29
Donga	46	29	25
Alibori	33	48	20
Borgou	45	35	19
Littoral	59	29	12
Wealth index quintile			
Poorest	24	25	51
Poorer	25	33	42
Middle	26	39	35
Richer	37	41	22
Richest	64	30	7
Age of respondent			
16–24	36	36	28
25–34	39	34	28
35–44	36	34	30
45–54	34	33	34
55–64	33	33	34
65+	31	31	37
Sex of respondent			
Male	35	34	31
Female	37	33	30
Religion of respondent			
Other traditional	27	23	50
Traditional (Vodoun)	27	30	42
Other religion	25	34	41
Celeste	29	35	37
No religion	35	31	34
Protestant methodist	31	35	34
Other Protestant	38	32	31
Other Christian	33	37	30
Catholic	42	33	25
Islam	40	37	23
Ethnicity of respondent			
Betamari and related	32	22	47
Fon and related	32	34	34
Yoruba and related	35	34	32
Yoa and Lokpa related	39	31	30
Peulh and related	24	46	29
Adja and related	43	30	27
Countries bordering Benin	53	24	23
Other countries	50	29	21
Bariba and related	40	42	18
Other ethnic group	47	36	17
Dendi and related	54	30	16
Education of respondent			
No education	30	33	38
Primary	37	36	28
Secondary 1	46	35	19
Secondary 2	60	30	10
Higher	64	29	7

*Source*: Calculated from Benin DHS 2006

There appears to be little difference between most age groups, although older respondents (aged 65+ years) do have higher than expected prevalence rates of 4+ deprivations. Given data were collected at household level we cannot comment or assess the extent of intra-household poverty or inequity or comment on gender differences in poverty rates. That said, such issues could be addressed in further work, which would employ an individual-level questionnaire, as has recently been done in the UK 2012 Poverty and Social Exclusion Survey. In terms of ethnicity, it appears respondents from Betamari and related groups are worse off than their compatriots, while those from Dendi and Bariba groups experience relatively low rates of 4+ deprivation. The expected relationship between education and multiple deprivation is confirmed, with a clear gradient apparent (i.e. those with no education have far higher rates of 4+ deprivation).

## Adaptive Preferences

One issue which arises for studies which examine people’s subjective opinions about their own social position, wellbeing or welfare is that of adaptive preferences. Briefly put, this theory posits that poor or deprived people may lower their expectations of what they might otherwise be entitled to (e.g. to receive an education, to gainful employment, to health care when sick and support in times of need), and these lower (or bounded) horizons effectively underplay what they think are the necessities of life in a given society. In other words, according to Nussbaum,

people’s desires and preferences respond to their beliefs about norms and about their own opportunities. Thus people usually adjust their desires to reflect the level of their available possibilities….People from groups that have not, persistently, had access to education, or employment outside the home, may be slow to desire these things because they may not know what they are like or what they could possibly mean in lives like theirs (Nussbaum 1999: 11).

People experiencing poverty (and deprivation), then, are effectively discouraged from demanding radical change or high enough norms or standards to meet expert opinions (or peoples’ needs), and instead accept their circumstances out of necessity (Sen 1992). This, some claim, is a potential source of bias, in that it results in a constrained expression of what social norms really are or should be, and thus renders them unreliable.

Adaptive preferences have been well studied by researchers, and varying degrees of evidence of this phenomenon have been produced. Examples include Burchardt (2004) who used British Household Panel Survey data to identify and quantify the process of adaptation with regards changes in income and satisfaction with household income. She found (unsurprisingly) that people experiencing a fall in income from one year to another were less satisfied than those who had a steady income, but also that those individuals who experienced an increase in income were also less satisfied. Burchardt notes

This suggests that income is a poor proxy for satisfaction but it does not provide firm evidence for the existence of adaptation over the short term. Over a longer period, those who have experienced falling incomes are less satisfied than those who have had constant income, while those who have experienced rising incomes are no more satisfied than those who have had constant incomes. This suggests that over a longer period, adaptation to changes in income is asymmetric: people adapt to rising incomes but less so falling incomes (Burchardt 2004: iv).

Halleröd using data from Sweden investigated whether people adapted their consumption preferences to fit their ability to consume (Halleröd 2006). One assumption tested was that the fewer economic resources a person had (i.e. their level of income), the fewer consumption items they would deem as desirable, reflected by an increase of ‘do not want’ answers as economic resources decrease. While he found that “*people with low income seem to adapt their preferences, being content with less*” (2006: 386) the evidence was “*admittedly weak*”. There was stronger evidence that long-term economic constraints encouraged adaptation of preferences, reflected by the fact that people with limited access to economic resources were more likely to say they ‘cannot afford’ different kinds of consumption items, although they were also more likely to say they ‘do not want’ to consume various items. Halleröd concluded

The analysis also indicates that, in line with the theoretical assumption, the longer a difficult economic situation lasts, the more people adjust their aspirations. Hence, it would seem that people adapt their preferences in relation to their economic circumstances, and the interpretation here is that they do so in order to escape the unpleasant feeling of S-RD (Subjective Relative Deprivation) (2006: 388).

Crettaz and Suter (2013) using data on Switzerland, recently found evidence of adaptive preferences (downward adaptation) among individuals affected by income poverty, and that both indicators of material deprivation and subjective indicators related to income satisfaction and financial constraints were affected. However, they note, “*bias caused by adaptation processes…varies considerably among the various measures and indicators examined within each of these two groups, and some of them, in fact, appear not to be affected at all*” (2013: 148). They found that overall deprivation indexes like those developed by Townsend (1979) and Halleröd (1995) were either only weakly affected or did “*not show any bias due to adaptive preferences of poor people*” (Crettaz and Suter 2013:149).

Noble et al. (2008) examined whether adaptive preferences and bounded realities affected a democratically defined measure of poverty in South Africa. Such an exercise is particularly important in a country like South Africa, with its legacy of Apartheid which systematically marginalised and disadvantaged a majority of the population. Any attempts at a “democratic” definition of poverty, using the consensual approach, would need to ensure that what emerged had not been dampened down by peoples’ experiences. Controlling for differences between population groups and area (i.e. urban/rural), Noble et al. found that respondents located above a subjective poverty line (i.e., who reported that their household income was greater than the amount required to make ends meet) were more likely than those who were not above the subjective poverty line to define certain items (e.g. ‘meat/fish/vegetarian equivalent every day’) as essential. A recent paper by Wright and Noble (2013) explored the issue of adaptive preferences in South Africa in more detail, and examined whether or not respondents possessing an item were more or less likely than those lacking it to consider it a necessity. While respondents who owned items were more likely to claim it to be necessary, what was also apparent was the fact that those lacking the item did so because they could not afford it, not because they did not want it. So while they found some evidence of adaptive preferences, with poor people less likely to report an item as a necessity, they nonetheless argue convincingly that this does not affect the reliability of the method or its applicability to low income countries.

**Fig. 4:**
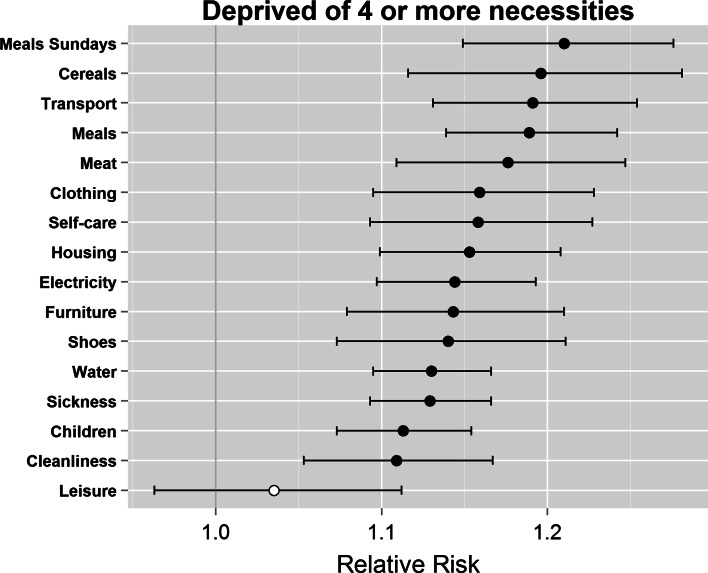
Relative risk ratios for respondents deprived of 4+ items thinking items on the deprivation index as ‘essential’, compared to respondents with no deprivations. (Color figure online)

We used the Benin data to see if there was any evidence of adaptive preferences among people experiencing poverty. If it could be shown that respondents who report their needs for a particular item were “*not satisfied at all*” (i.e. were deprived of a socially perceived necessity, see Table 1) were systematically *less* likely than those who felt their needs were met (and thus not deprived) to consider items on the deprivation index as essential, this would suggest some evidence of adaptive preferences, with implications for the overall deprivation index. Controlling for respondent’s age, sex, place of residence (i.e. urban or rural location) and household wealth quintile, we compared the relative risks of those deprived/not deprived thinking whether an item was essential or not (Fig. 4). It is apparent that in the case of Benin, people in poverty (i.e. defined here as those deprived of four or more socially perceived necessities) were marginally more likely than those not experiencing any deprivations (i.e. those not in poverty) to consider all of items on the deprivation index essential. This analysis provides evidence to contest the claim that poor people adapt their preferences downwards with regards what they consider the necessities of life, and suggests instead that they in fact have opinions and views very similar to the non-poor as to what constitutes a decent standard of living.

To confirm that this result was not due to the use of the overall deprivation index, risk ratios were run for each individual item on the index, comparing those reporting that their needs were not met at all (i.e. and were thus deprived) with those whose needs were met (i.e. who were not deprived). In almost every instance (i.e. 254 out of 256 tests^10^), deprived respondents were significantly *more* likely to consider each of the items in the deprivation index to be essential, compared to those not deprived (see charts in Appendix “4”).

This is not the first time a study of consensual poverty has reached such a conclusion. Gordon and Pantazis (1997b) found no major differences between “*multiply deprived*” and “*less deprived*” respondents about the necessity of different items; in fact, respondents who considered themselves “*genuinely poor all the time now*” were more likely to report certain items and activities as necessary than *never*-*poor* respondents, including having carpets in living rooms and bedrooms, having a television and being able to have a night out fortnightly. Nevertheless, it should be noted that the differences between the two groups are extremely small: whether controlling for age, sex, place of residence and wealth or not, deprived respondents are only about 10 % more likely to perceive items as necessities than the non-deprived ones. This however represents strong evidence of lack of adaptive preference among the surveyed population of Benin.

## Discussion

The aim of this paper was to demonstrate how the consensual approach can be effectively used to produce a valid and reliable index of deprivation for a low income country. Despite considerable socio-economic and cultural variations in Benin, with different language, religious and ethnic groups, it was shown that clear consensus exists about what elements constitute the basics of a decent standard of living, which no one should lack. In doing so, it is clear that people’s conceptions of what constitute basic needs and poverty go well beyond narrow conventional definitions (e.g. sufficient money to cover minimal dietary needs), which are commonly reported on using money-metric indicators. The consensual approach allows us to expand on Townsend’s concept of relative deprivation and exclusion from customary norms, to reveal, even in situations of widespread considerable want and lack, the higher aspirations which people expect to meet or be met. A number of rich countries, including member states of the European Union, have moved away from minimal definitions of poverty, and increasingly incorporate people’s attitudes about societal-deemed norms and necessities (both material and social) in their measures and estimates of poverty. As such, the consensual approach to poverty research looks set to stay, and in time will form the basis of poverty assessments in many more countries.

To date, there have been no concerted efforts to run comparable consensual studies in low and middle income countries. The DHS programme, with its already established survey infrastructure, presents an ideal mechanism through which poverty modules similar to the one used in this paper (for which the original questionnaire is provided in Appendix “1”) might be used across a number of developing countries. Other cross-national survey programmes, like UNICEF’s Multiple Indicator Cluster Surveys (MICS)^11^ with their focus on children’s needs, the World Bank Living Standard Measurement Surveys (LSMS) or the Global Barometer Surveys^12^ would also be a possible way to gather comparable data, with standardised wording for questions, item lists and response categories. The resulting analyses might demonstrate (or confirm what is already known in Europe, thanks to the Eurobarometer Survey) that people tend to agree on what the necessities of life are and what constitutes a decent standard of living, from which no one should be excluded. The accuracy and policy relevance of poverty measures can be greatly enhanced if the views of the population (and particularly the ‘poor’) are incorporated into the measure of poverty. There are different ways to do this, such as the use of focus groups, incorporating nationally or internationally agreed standards into the measure (such as minimum standards and/or rights of access to education, water quality, housing quality, etc. found in the constitutions of some countries—such as South Africa). The approach taken in this paper follows the ‘consensual’ or ‘perceived deprivation’ approach to measuring poverty by investigating the public’s perceptions of minimum needs through a representative survey.

Evidence was provided to contest the position that adaptive preferences can limit the effectiveness of the consensual approach, as poor people are more likely to underplay the importance or necessity of items considered essential or necessary for a decent standard of living. In Benin, not only was consensus clear across all social groups about the importance of all items, but with regards the items in the final deprivation index, people experiencing poverty were more likely to consider each of the items as essential than people not in poverty. As Wright and Noble (2013) found in South Africa, lacking or not possessing an item did not necessarily mean respondents did not aspire to having it; rather, in most instances people lacked items because they could not afford them not because they did not want them. As such, the consensual, or socially perceived necessities, approach looks to be a valid and reliable method for examining poverty in low income countries. Using it to develop measures or indicators for different sub-groups (e.g. specifically for children or the elderly) as has been done in Europe could form the basis of much important work in the future.

## Figures and Tables

**Table 5:**
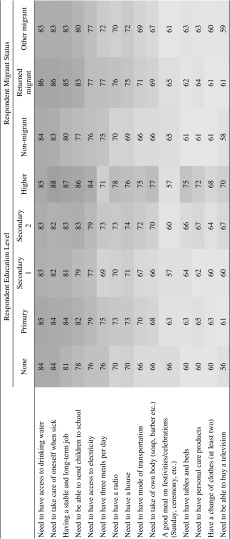

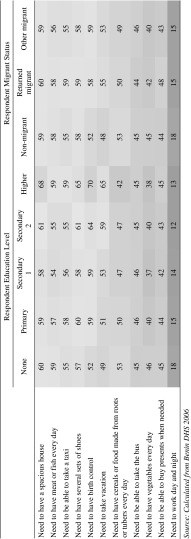
Heat map of attitudes to items considered “essential”, by respondent education level and migrant status (%). (Color figure online)

**Table 6:**
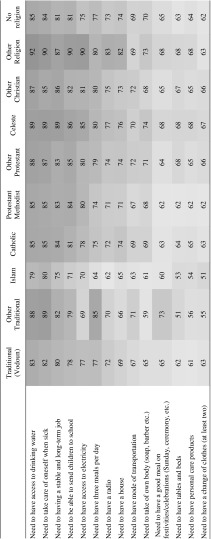

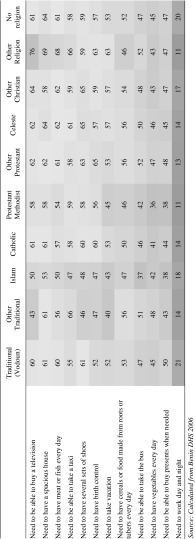
Heat map of attitudes to items considered “essential” by respondent religion (%). (Color figure online)

**Table 7:**
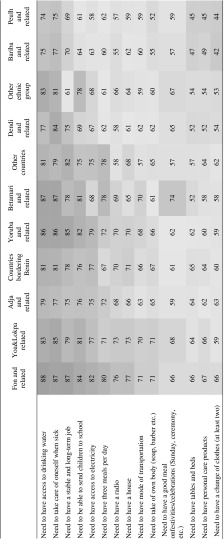

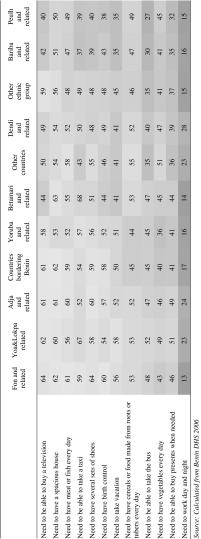
Heat map of attitudes to items considered “essential”, by respondent ethnic group (%). (Color figure online)

## Appendix 2: Heat maps to demonstrate consensus

See Tables 5, 6 and 7.

## Appendix 3: Item validation

**Table:**

	Difficult compared to good or more or less OK (relative risk with 95 % CIs)
Validator 1—evaluation of household income status	
Number of meals every day	14.7 (10.8–20.1)
Consumption of cereals and tubers every day	9.4 (6.9–12.8)
Clothing	6.3 (5.0–7.9)
Shoes	6.0 (4.8–7.5)
A good meal on festivities/celebrations (Sunday, ceremony, etc.)	5.7 (4.3–7.5)
Consumption of meat or fish every day	5.6 (4.5–6.9)
Cleanliness/personal hygiene	4.1 (3.4–5.1)
Housing	3.8 (3.3–4.4)
Care in case of sickness	3.6 (3.1–4.3)
Self-care products in the house	3.5 (3.0–4.1)
Education for children	2.8 (2.3–3.2)
Availability of transport	2.5 (2.3–2.7)
Availability of leisure	2.2 (2.0–2.5)
Furniture in the house	2.2 (2.0–2.4)
Availability of drinking water	1.9 (1.7–2.1)
Availability of electricity	1.6 (1.5–1.7)

	Goes into debt compared to able to save (relative risk with 95 % CIs)
Validator 2—current financial situation	
Number of meals every day	8.3 (6.0–11.5)
A good meal on festivities/celebrations (Sunday, ceremony, etc.)	7.6 (5.3–10.9)
Consumption of cereals and tubers every day	7.6 (4.9–11.8)
Consumption of meat or fish every day	6.3 (4.8–8.1)
Shoes	5.4 (4.1–7.1)
Clothing	5.1 (4.0–6.7)
Cleanliness/personal hygiene	3.7 (3.0–4.7)
Care in case of sickness	3.2 (2.7–3.8)
Education for children	3.1 (2.5–3.8)
Housing	3.0 (2.6–3.6)
Self-care products in the house	2.9 (2.5–3.5)
Availability of leisure	2.5 (2.1–2.8)
Availability of transport	2.3 (2.1–2.6)
Furniture in the house	2.2 (2.0–2.4)
Availability of drinking water	1.8 (1.6–2.1)
Availability of electricity	1.6 (1.5–1.7)

	Very unstable compared to stable (relative risk with 95 % CIs)
Validator 3—stability of family income	
Number of meals every day	3.2 (2.4–4.4)
Consumption of cereals and tubers every day	2.9 (2.0–4.2)
A good meal on festivities/celebrations (Sunday, ceremony, etc.)	2.8 (2.1–3.8)
Consumption of meat or fish every day	2.8 (2.3–3.4)
Clothing	2.7 (2.2–3.5)
Shoes	2.6 (2.1–3.3)
Housing	2.2 (1.9–2.5)
Self-care products in the house	1.8 (1.6–2.0)
Care in case of sickness	1.8 (1.6–2.0)
Education for children	1.8 (1.6–2.0)
Availability of transport	1.7 (1.6–1.9)
Cleanliness/personal hygiene	1.7 (1.4–2.0)
Furniture in the house	1.6 (1.5–1.7)
Availability of leisure	1.6 (1.4–1.7)
Availability of drinking water	1.5 (1.3–1.6)
Availability of electricity	1.4 (1.3–1.5)

	Bottom quintile compared to top quintile (relative risk with 95 % CIs)
Validator 4—asset index quintiles	
Availability of electricity	9.8 (8.3–11.6)
Shoes	7.5 (5.7–9.8)
Self-care products in the house	7.2 (5.6–9.2)
Clothing	7.0 (5.4–9.1)
Cleanliness/personal hygiene	6.9 (5.3–9.1)
Consumption of meat or fish every day	6.9 (5.2–9.0)
A good meal on festivities/celebrations (Sunday, ceremony, etc.)	6.8 (5.0–9.2)
Education for children	6.0 (4.8–7.4)
Care in case of sickness	5.6 (4.6–6.7)
Availability of drinking water	5.0 (4.2–6.0)
Number of meals every day	4.9 (3.7–6.6)
Furniture in the house	4.5 (3.9–5.2)
Consumption of cereals and tubers every day	4.1 (3.0–5.5)
Housing	3.7 (3.1–4.3)
Availability of transport	2.9 (2.5–3.2)
Availability of leisure	2.8 (2.4–3.3)

## Appendix 4: Relative risk ratios comparing the opinions of deprived versus non-deprived respondents about whether items in the 16-item deprivation index were essential

*Note*: These charts show the relative risk of respondents deprived of the item in the heading thinking items on the deprivation index to be essential, compared to those not-deprived of items in the heading. RRs to the right of the line set at 1 imply deprived respondents are more likely to think items on the Y-axis to be essential; where error bars do not touch or cross 1, there is a statistically significant difference between deprived and non-deprived thinking an item is essential.

## Footnotes

1
www.socialprotectionfloor-gateway.org/.

2 This raises interesting questions about potential differences in the interpretation of key concepts; however, the consistency of consensus across all ethno-linguistic groups suggests widespread understanding of the key concepts of items/activities considered essential or necessary for a ‘vie correcte’.

3 Available at http://measuredhs.com/publications/publication-FR197-DHS-Final-Reports.cfm, accessed 16 November, 2012.

4 The question asked, in French, was "*Estimez-vous indispensable, nécessaire ou non que les items suivants font partie des besoins minima pour avoir une condition de vie correcte*?".

5 This is an English translation; the original questionnaire for this poverty module is provided in Appendix 1.

6 The question asked, in French, was “*Etes-vous satisfait par rapport aux besoins minima de votre ménage dans les domaines suivants*?”. No details are provided as to why follow up questions are asked of this particular subset of questions.

7 Mack and Lansley’s work also used the single survey format.

8 In other words vertical agreement on the relative importance of say, access to water compared to being able to take a vacation.

9
http://databank.worldbank.org/data/views/reports/tableview.aspx# , accessed 16 December, 2013. Benin poverty lines available at www.microfinance.com/English/Papers/Scoring_Poverty_Benin_EN_2010.pdf.

10 Relative risk ratios for each item were run against whether or not respondents were deprived/not deprived of the remaining 15 items. 16 × 16 results in 256 separate tests.

11
www.childinfo.org/mics.html, accessed 7 December, 2013.

12
www.globalbarometers.org/ , accessed 7 December, 2013.
